# How can communication to GPs at hospital discharge be improved? A systems approach

**DOI:** 10.3399/BJGPO.2021.0148

**Published:** 2021-12-08

**Authors:** Nicholas Boddy, Stephen Barclay, Tom Bashford, P John Clarkson

**Affiliations:** 1 GP, Radcliffe-on-Trent Health Centre, Nottingham, UK; 2 Research Collaborator, University of Cambridge Engineering Design Centre, University of Cambridge, Cambridge, UK; 3 Visiting Researcher, Primary Care Unit, Department of Public Health and Primary Care, University of Cambridge, Cambridge, UK; 4 GP and Honorary Consultant Physician in Palliative Care, Cambridge, UK; 5 Honorary Professor of Palliative and Primary Care, University of East Anglia, Norwich, UK; 6 University Senior Lecturer in General Practice and Palliative Care, Primary Care Unit, Department of Public Health and Primary Care, University of Cambridge, Cambridge, UK; 7 Research Fellow, NIHR Global Health Research Group on Neurotrauma, Cambridge, UK; 8 Clinical Lecturer and Honorary Specialist Registrar in Anaesthesia, Division of Anaesthesia, University of Cambridge, Cambridge, UK; 9 Clinical Lecturer, University of Cambridge Engineering Design Centre, University of Cambridge, Cambridge, UK; 10 Professor of Engineering Design, Director of the Cambridge Engineering Design Centre and Co-Chair of Cambridge Public Health, University of Cambridge, Cambridge, UK; 11 Professor of Healthcare Systems, Faculty of Industrial Design, Delft University of Technology, Delft, The Netherlands

**Keywords:** primary–secondary care interface, interprofessional communication, communication, patient safety, systems approach, service improvement, patient discharge

## Abstract

**Background:**

Poor communication to GPs at hospital discharge threatens patient safety and continuity of care, with reliance on discharge summaries that are commonly written by the most junior doctors. Previous quality improvement efforts have largely focused on adherence to standardised templates, with limited success. A lack of understanding has been identified as a cause of the issue’s resistance to decades of improvement work.

**Aim:**

To understand the system of communication to GPs at hospital discharge, with a view to identifying potential routes to improvement.

**Design & setting:**

A qualitative exploration of the secondary-to-primary care communication system surrounding a large UK hospital.

**Method:**

A systems approach, recently defined for the healthcare domain, was used to structure and thematically analyse interviews (*n* = 18) of clinical and administrative staff from both sides of the primary–secondary care interface, and a subsequent focus group.

**Results:**

The largely one-way communication system structure and the low level of hospital stakeholder insight into recipient GP needs emerged as consistent hindrances to system performance. More open lines of communication and shared records might enable greater collaboration to share feedback and resolve informational deficits. Teaching sessions and assessments for medical students and junior doctors led by GPs could help to instil the importance of detail and nuance when using standardised communication templates.

**Conclusion:**

Facilitating the sharing of performance insights between stakeholder groups emerged as the key theme of how communication might be improved. The empirical measures proposed have the potential to mitigate the safety risks of key barriers to performance such as patient complexity.

## How this fits in

Patients continue to come to harm at hospital discharge owing to suboptimal communication. Decades of improvement work have not yet achieved satisfactory success. A systems approach revealed that the largely one-way communication system leads to a disconnect between primary and secondary care stakeholders, with a lack of insight into recipient GP needs. A more collaborative approach to discharge communication with greater input by GPs is required to establish a more operant mode of system learning. Proposals are presented to enable sharing of insights and mitigate key performance barriers such as patient complexity and overstandardised communication.

## Introduction

Preventable patient harm during the transition of care at hospital discharge has been acknowledged as an ongoing problem, both in the UK^1,2^ and internationally.^3^ Harm has often been found to relate to communication,^4,5^ specifically to the patient’s GP,^6,7^ and can lead to adverse events such as hospital readmissions^8^ and deaths.^9,10^ This communication occurs almost exclusively through a contractually enforced discharge summary.^11,12^ Over 90%^13–15^ of discharge summaries are authored by newly qualified foundation doctors, who struggle to analyse the information to include^16^ and lack insight into the importance of communication for the recipient GP.^17–20^ Discharge summaries are frequently written under high workload pressures, which reduce their quality.^19,21,22^ Poor quality communication significantly impacts recipient GPs, hampering their ability to make clinical decisions^23^ and adding to their already significant workload.^24^ Financial incentives and subsequent contractual obligations^25^ for electronic delivery of discharge summaries within 24 hours^12^ of discharge have improved the speed of provision,^26^ but not the quality of content.^27^

Work on discharge communication quality has largely revolved around standardisation, led nationally in the UK by the Professional Record Standards Body (PRSB).^28^ Adherence to these standards has been the primary outcome measure of most published studies in this area, but success has been variable.^13,14,29^ Many of the metrics used poorly describe subjective components,^13,30^ such as the 'description of symptoms', and are unlikely to measure the detail that can be so critical to GPs,^31^ especially for complex, multiply-comorbid and older patients, who are at particularly high risk of poor communication.^6,32,33^ Despite suggestions that these expanding^34^ patient groups^35,36^ should receive greater attention in the communication process, published guidance is limited in scope, consisting mainly of simple recommendations to make supplementary telephone calls to the GP.^22,37,38^

The breadth of caseload and differing opinions on communication between GPs and hospital doctors^39–41^ make the quality of this communication difficult to consistently define and measure. This is arguably owing to a lack of understanding of this complex problem as Markiewicz *et al* ^6^ have recently described and, given the issue’s ‘resistance’ to decades of work, the emergent question is therefore *how* can communication to GPs at hospital discharge be better understood and improved?

## Method

**Figure 1.**
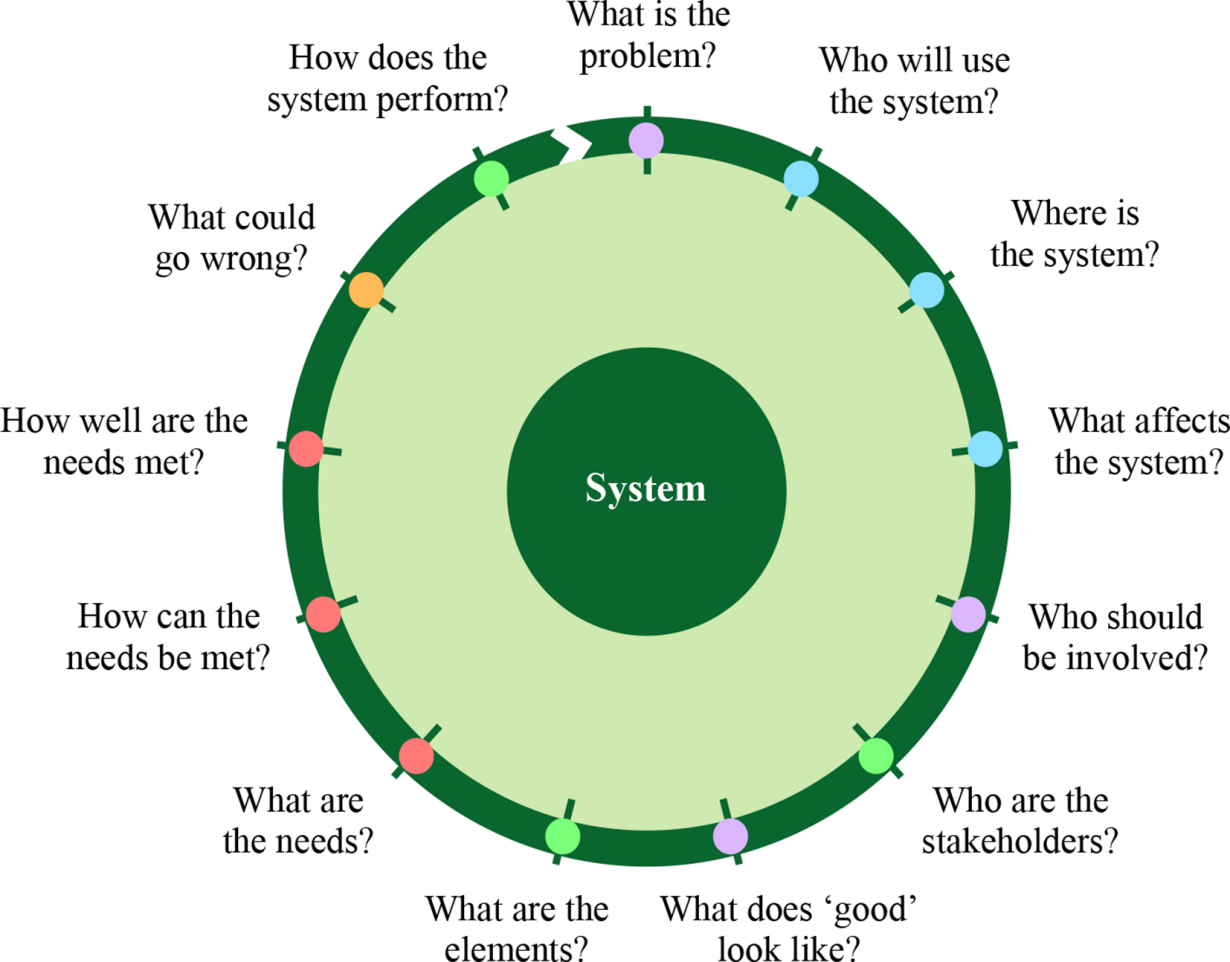
A systems approach framed as an iterative series of questions. Blue = people perspective. Green = systems perspective. Red = design perspective. Orange = risk perspective. Purple = systems approach ‘project questions’. *Non colour-dependent versions of all figures are available in the supplementary materials, under the Figures & Data tab.*

To understand how improvement might be achieved, the conceptual framework of a systems approach, as recently defined by the Royal College of Physicians and Royal Academy of Engineering,^42^ was used to qualitatively explore the system of discharge communication around a large tertiary hospital in England. This approach has been championed for tackling complex and ‘messy’ problems, drawing on the experiences of systems engineering, design theory, and healthcare quality improvement. Its four perspectives of 'people', 'systems', 'design', and 'risk' are incorporated into an iterative spiral of questions that serve to refine understanding of a problem (Figure 1) to respond to an improvement research question. Using this framework, the views of a variety of stakeholders were explored qualitatively (Table 1).

**Table 1.** Study design and sampling

Semi-structured interviews of clinicians (*n* = 10)
**Stakeholder type**	**Number**	**Notes**
FY1 doctors	2	Based in geriatrics
Hospital registrars	1	Based in geriatrics
Consultants	3	2 geriatricians, 1 consultant physician in senior management position
GP registrars	2	1 in first year of training, 1 in final year of training
GPs	2	1 in early career (<5 years post-qualification), 1 in later career (>20 years since qualification)
**Focus group of clinicians** (*n* = 1)*The group comprised:*		
FY1 doctors	1	Based in geriatrics, not previously interviewed
Consultants	2	2 geriatricians, one previously interviewed
GP registrars	2	Both in final training year, one previously interviewed
GPs	1	In later career, previously interviewed
**Semi-structured interviews of admin and supporting staff** (*n* = 8)		
**GP surgery admin staff** (*n* = 5)*Five GP surgeries were sampled purposively through interview with a single staff member. These surgeries were stratified by the following characteristics:*		
Size of surgery	2 multisite, 3 single-site	
Location type	4 within city perimeter, 1 rural	
IT System	4 SystmOne, 1 EMIS	
**Hospital staff** (*n* = 3)*Participants were identified as the project progressed and recruited by convenience sampling. Participating staff (1 per setting) were from: (i) the GP liaison office, (ii) the GP pharmacy queries office, and (iii) the IT development and support team*		

FY1 = Foundation Year 1.

### Data collection

Doctors, at all levels of seniority, in hospital and in general practice are respectively the primary authors and recipients of discharge letters. Their perspectives were therefore identified as a key focus for this scoping work, and clinicians thus formed the majority of study participants as outlined in Table 1. To gain a wider understanding of the context of communication, administrative and infrastructural staff on both sides of the primary—secondary care interface were included as an additional scoping dataset, as stratified in Table 1. Time and resource constraints prevented inclusion of patients and wider groups of stakeholders. All participants were recruited purposively by convenience snowball sampling, through hospital departmental staff and the local GP training scheme, seeking a maximum diversity sample of seniority in both settings. A focus group of clinicians was held after the completion of the individual interviews. This was to collaboratively explore and further develop themes arising from initial semi-structured individual interviews, with previous interviewees invited to attend to give continuity to the study. A focus group size of six participants was chosen to balance contribution opportunities with a breadth of stakeholder viewpoints. Topic guides for the interviews and the focus group (Appendix A) were designed to address the systems-approach questions (Figure 1), with additional foci on complexity, perceptions of the patient perspective, and issues emerging during the study. Audio-recordings were taken of each interview (duration 30–45 minutes) and the focus group (duration 1 hour), with written consent. All interviews and the focus group were conducted by NB in private in offices or seminar rooms. TB attended the focus group as an observer and research assistant.

### Analysis

Given the focus of the study on the perspectives of doctors as the main initiators and recipients of discharge letters, analysis was primarily focused on the clinician dataset. Digital audio-recordings of the clinician interviews and the focus group were transcribed verbatim by NB. Recordings of the administrative and infrastructural staff interviews were not transcribed, instead detailed reflective notes were written after repeated reviews of the audio-recordings. Thematic analysis of the transcribed interviews and focus group and reflective notes was carried out in NVivo qualitative data analysis software, using the constant comparative method in a primarily inductive manner. The resultant themes were then clustered by their ability to address the systems-approach questions (Figure 1), allowing inductive codes to be applied to a pre-existing model. Coding was performed on the first clinician interview jointly with TB, while the remaining nine clinician interviews and focus group were coded by NB alone.

## Results

Results are presented in relation to the systems-approach questions (Figure 1) and grouped to form a cohesive narrative. The perspectives the questions bring are denoted in brackets. Participant verbatim quotations are presented in italics.

### Where is the system? What are the elements? (Systems and people perspectives)

**Figure 2.**
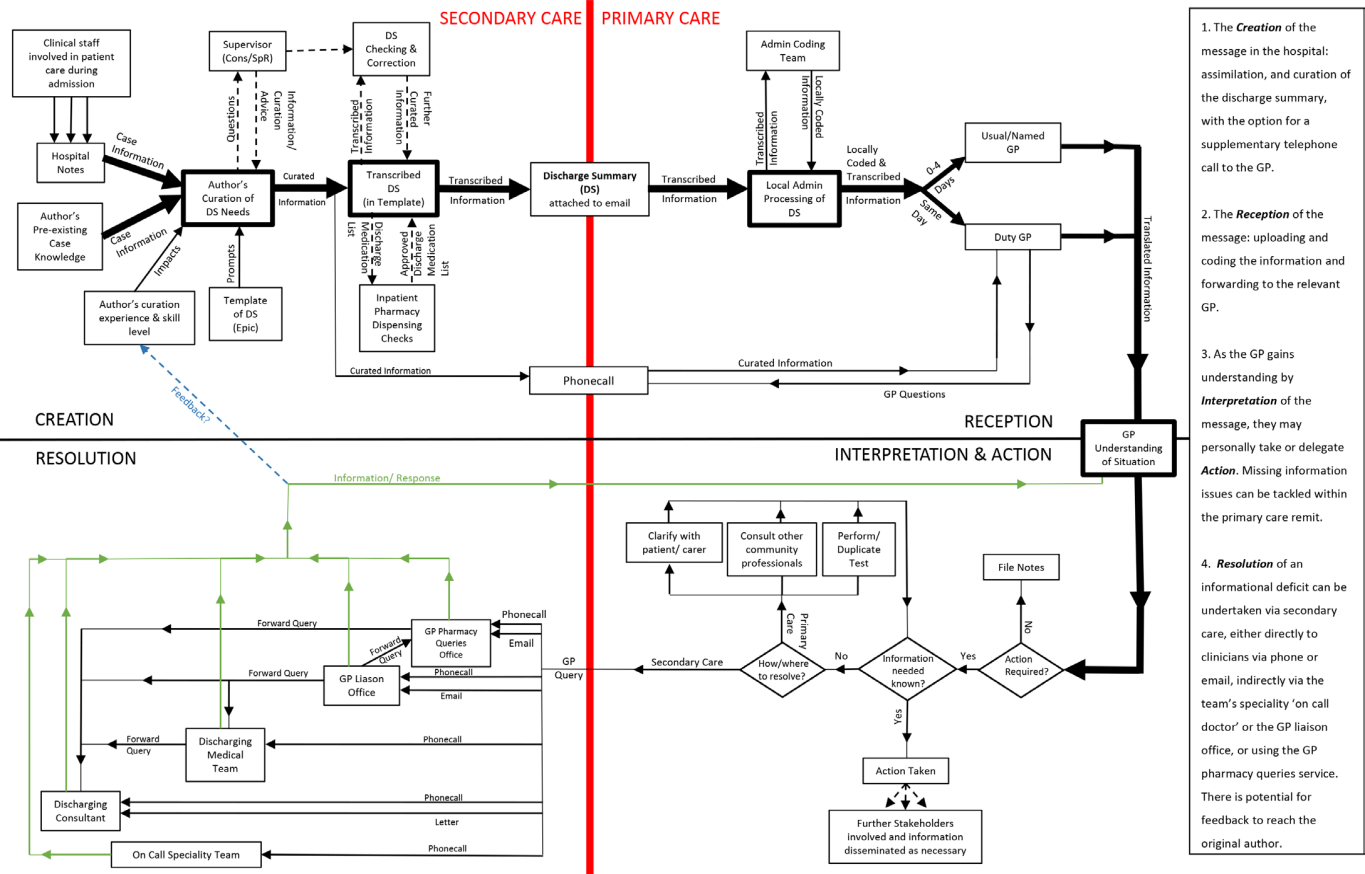
A model of the local architecture of the current system of communication to GPs at hospital discharge, constructed as a hybrid of descriptions provided by the study participants and shown as four phases. Dotted lines represent optional elements to the communication system. *Zoom-enabled and* *non colour-dependent versions of all figures are available in the supplementary materials, under the Figures & Data tab.*

Figure 2 represents a generalised system model of the local discharge communication system between secondary and primary care, constructed as a hybrid and amalgamation of participant descriptions. The system falls broadly into four phases. The number of system elements, stakeholders, and quantity of information to be communicated can rapidly increase as a patient develops *'multiple strands of care needs'* and consequent *'multiple strands of interactions with healthcare professionals'* (GP registrar 1) during the admission and after discharge. As patient care becomes more multidisciplinary, the system becomes inherently larger and more complex.

### Who are the stakeholders? Who will use the system? (Systems and people perspectives)

The list of potential system users, as a subset of the system’s stakeholders, emerged to include all staff involved in patient care during the discharge period, owing to their potential need to transmit or receive information. As well as the key system using stakeholders outlined in Table 2, additional key stakeholders of note included: the GP pharmacy queries office (hospital based), which fields and answers discharge medication queries from hospital letters, contacting clinical teams if necessary; the GP liaison office (hospital based), which passes non-urgent GP queries onto the relevant hospital staff; and GP surgery administrative staff who receive incoming communications, passing them onto the relevant GP.

**Table 2.** Key system: using stakeholders, their needs, and how well they are met

	**Stakeholder needs**	**How well are their needs met?**
Patients	To understand what has happened, what medications to take, and what is happening next For care to be provided in a coordinated manner where necessary	'*Probably the most common thing I find myself doing is helping them to understand what has been a very frightening period of time, with often quite poor communication about what’s going on* *.'* (GP 2) *'* *I think as a patient, I'd be very disappointed that the hospital were looking at letters that are just being generated, no one’s really sure what’s on it, the person who wrote didn’t know, we’re not sure if the GP practice is going to get it* *.'* (Consultant 2)
Hospital junior doctors(FY1 and SHO)	Time to write the discharge summary before discharge To know the relevant information to include in the discharge summary Support or advice when unsure Feedback on current performance and areas for improvement	*'* *Junior doctors, who might not know the patient well, who are trying to do a million and one other things, getting bleeped by someone to go and do something. This is not setting up a system where somebody’s likely to produce a good output* *.'* (Consultant 3) *'* *I don’t think I consistently know what a GP wants to know in a discharge summary* *.* *'* (FY1 doctor 2) *'* *Actually, I don't think we knew what we were doing* [when we were hospital juniors].*'* (GP registrar 2) *'* *Of course, I think they should ask for help more* *.'* (Geriatric registrar)
Hospital registrars	Discharge summaries to be done proficiently Discharge summaries to be done by junior doctors with support where required	*'* *If I had to go off and do a clinic full of* *30* *patients* *, and do central lines, I know I wouldn't be looking at the* *d* *ischarge* *s* *ummaries because I wouldn't have time* *.'* (Geriatric registrar) *'...* *there’s only been one time when the registrar was like* *"* *I'll look over this* *”* *, there’s not much oversight* *.'* (FY1 doctor 1)
Consultants	Discharge summaries to be done proficiently Other team members to author the discharge summaries with support if necessary Patients to be discharged as promptly as possible	*'* *It’s rare I see a* *d* *ischarge* *s* *ummary and think that’s exactly what I would like to be on it* *…* *it’s rarely going to go out as the quality you want it to be.* *'* (Consultant 2) *'* *You don't want to go up to the consultant and say* *"* *I'm really sorry* *…* *I'm trying to explain to the GP and it doesn't make sense* *"* *.* *'* (FY1 doctor 2)
GPsandGP registrars	Relevant and complete information for the patient, as quickly as possible Resolution of missing information in a timely manner with minimum additional workload	*'* *I've been working since August, and I genuinely have no idea what the quality of my summaries are* *.'* (FY1 doctor 3) *'* *I'd say things are improving, but I don't think we're consistently hitting that target of being good enough for a GP* *.'* (Geriatric registrar) *'* *I'm not on the receiving end of* *d* *ischarge* *s* *ummaries. So I don't know how well it works* *.'* (Consultant 1) [Do you feel like the system works?] *'* *Often around here, no* *.'* (GP 2) *'* *It works to a certain extent, I do get some information. There are definitely times when it’s lacking* *.'* (GP 1) [How easy is it to try and plug that information gap?] *'* *Usually pretty difficult* *.'* (GP 2)

FY1 = Foundation Year 1. SHO = senior house officer.

### What are the needs? (Design perspective)

**Figure 3.**
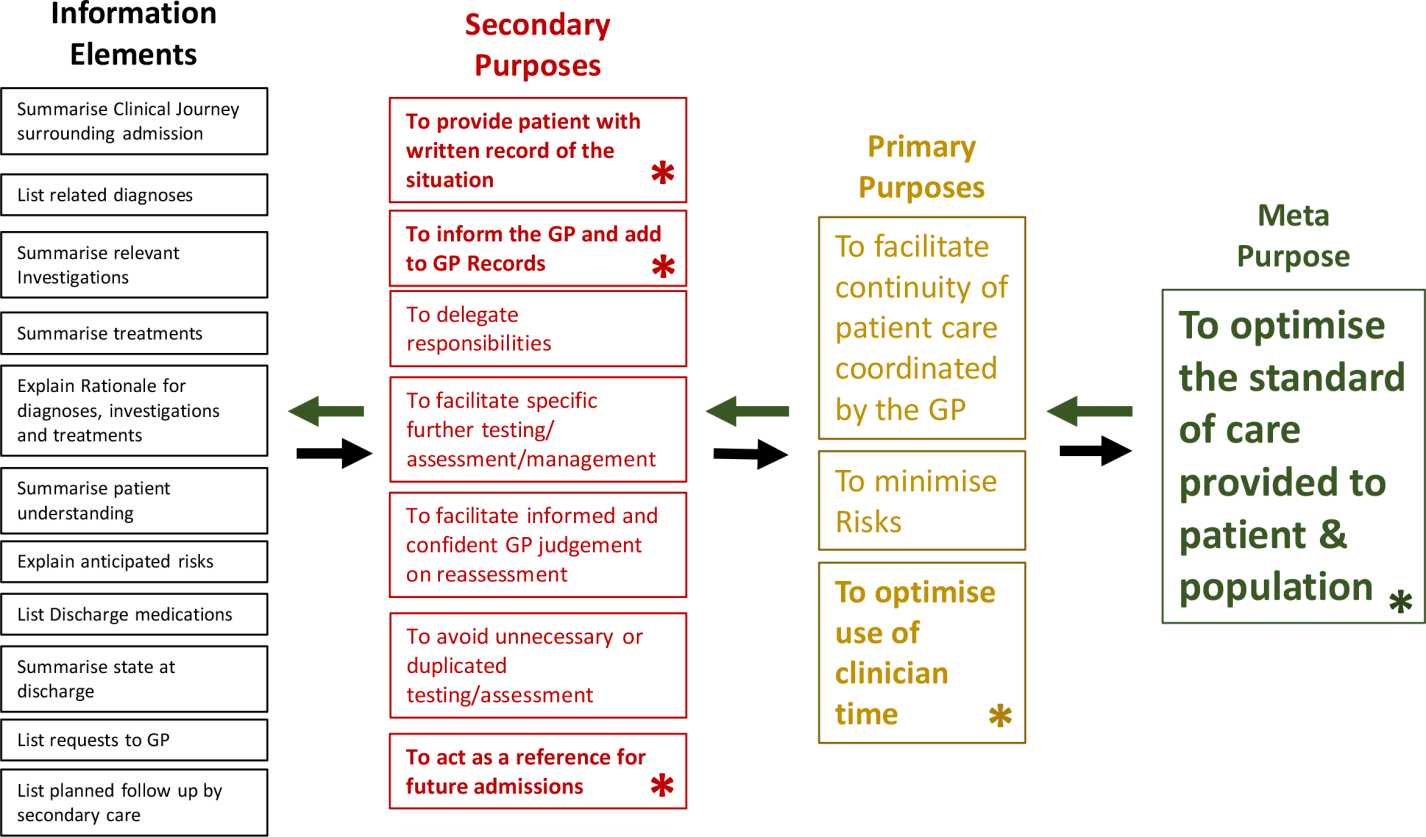
The purpose model. Clinical Information elements were found to serve specific purposes in an emergent sequence, shown as left to right. ‘Constant purposes’ that are always required are shown in bold and with asterisk, with others dependent on the patient involved. The right-to-left arrows indicate how purposes should be used to determine the information and detail within each element, as proposed for future education of discharge summary authors

A stakeholder’s needs are defined as the desired set of conditions for them to perform an activity. A system’s needs can be broken down to those of its stakeholders. Table 2 shows the needs of the key stakeholder groups, as described from their own perspectives and their views of the needs of patients. The relevance and completeness of information that secondary care should communicate with to meet GP needs was widely discussed in relation to specific elements of clinical information. All the elements that might be required were described similarly by at least one clinician in both primary and secondary care; these descriptions have thus been amalgamated and are represented as the left column of Figure 3. However, not all elements were deemed necessary for every patient, and excess information that would not be used was unanimously acknowledged as a negative feature. The need for specific elements was described to relate to the purpose they would be used for. These purposes, shown in Figure 3, were found to serve each other in an emergent sequence with the informational elements, as illustrated by the left-to-right arrows. Some purposes were deemed to always be required (shown in bold and with asterisk), while some were case dependent.

### How well are the needs met? How does the system perform? (Systems perspective)

Clinician participants offered their perceptions on how well the system performed to meet their own and each other’s needs, as well as the needs of patients. These are described in Table 2.

### What affects the system? What is the problem? (People, systems, and risk perspectives)

**Figure 4.**
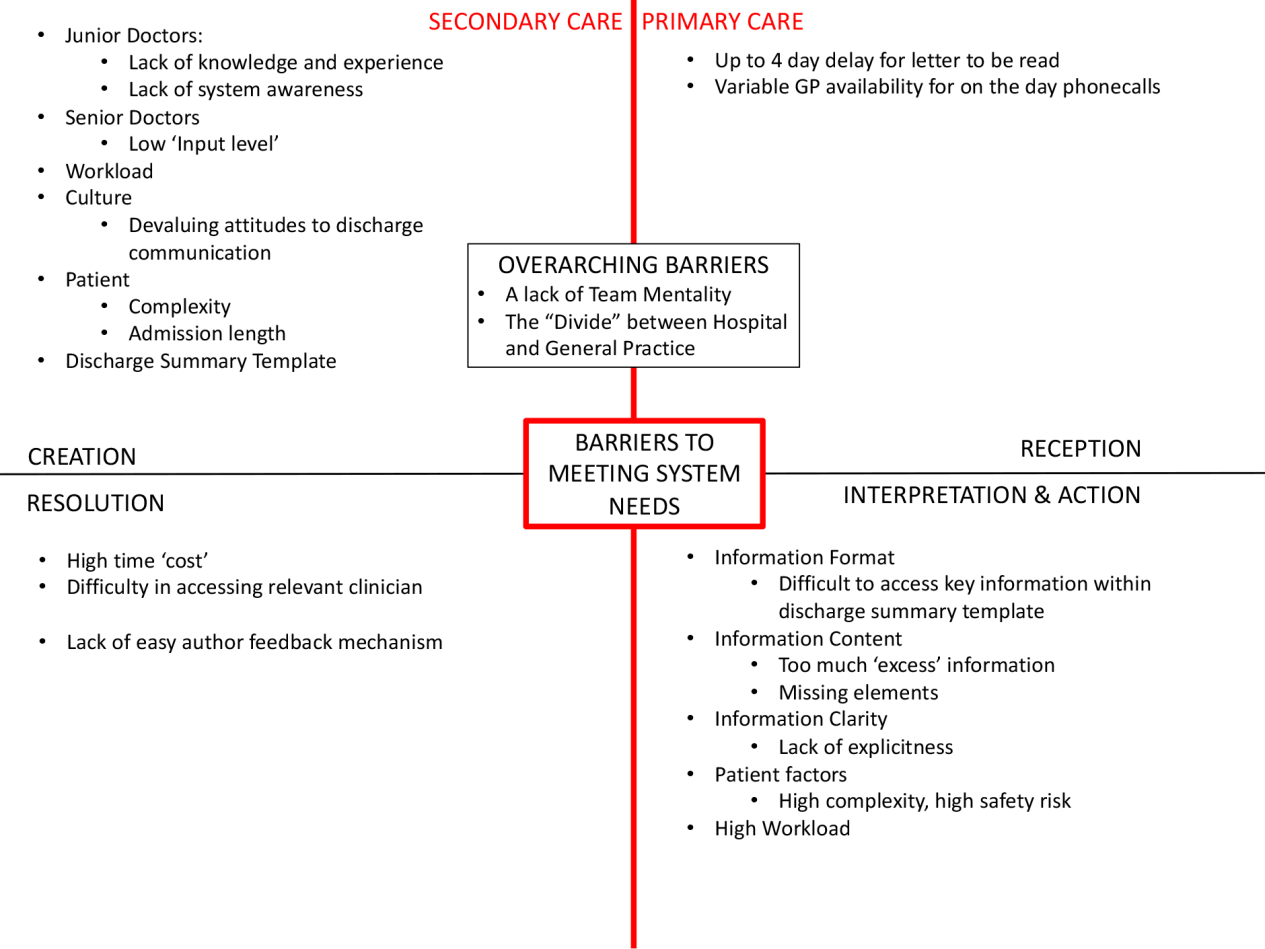
Barriers to system performance: themes from “'what affects the system?'” organised by system phase.

Several key themes were developed in answer to these questions, described below and illustrated in Figure 4, organised by system phase.

#### The experience level of junior hospital doctors and input from seniors

Junior doctors were described as less experienced, and this was seen to impact their technical medical knowledge and wider system awareness, potentially limiting both their level of understanding of the patient case involved, and their ability to describe it within a discharge summary.

*'* *I was required to write a discharge summary to explain what had happened. And I didn't really understand what had happened.* *'* (FY1 doctor 2)

Despite this, discharge summary writing was unanimously described as a job of the junior doctors and not a *'productive use of registrar or consultant time'* (consultant 3). Guidance from senior doctors was outlined as important by both consultants and the geriatric registrar, and although their input was generally described to be available, it emerged that junior doctors may still not ask for senior support, owing to *'a fear of being shamed'* (FY1 doctor 2) and a desire to appear competent. The skill of discharge summary curation was widely described as a process learnt by personal experience, rather than by formal teaching or supervision. This was despite feedback on the quality of discharge communication being described as rare or absent, particularly from GPs:

*'* *I don't think we were given any training whatsoever in terms of how to write a discharge summary, I think it was very much a trial by fire, learning.* *'* (FY1 doctor 1)

#### Presentation and completeness of information

The presentation of the information included in a discharge summary was described to heavily affect the ease and speed of *interpretation*. The discharge summary template was described as a hindrance to this process by both GPs and consultants, making the discharge summary *'very hard to read'* (GP 1). This issue was found to be commonly compounded by the *'noise'* (GP 1) of excess information, which the template often encouraged, while simultaneously hiding key aspects:

*'* *Because there’s so much of this extra information, and often the bits that you actually needed to know, are so buried in that* *.'* (GP 2)

Junior doctors generally saw the discharge summary template as a useful aid, although one also described potential limitations, particularly the idea of a standard template *'implies you don’t have to think as much as you should'* (FY1 doctor 2) with respect to the relevance of content.

The clarity and precision of discharge summary content were described to be critical to *'actually make care coordinated'* (consultant 4), particularly with elements of information that would support follow-up requests or any decision needing to be made by the GP. Significant lack of clarity (or completely missing information) was described as a common problem, and although primary care participants indicated they might still be able to interpret unclear information, this generated greater uncertainty and risk, gave potential for *'substandard care'* (GP registrar 2), increasing the need to use time-consuming *resolution* pathways.

#### Workload

Recognition was unanimously made of the time and workload pressures facing the entire system. The huge demand to *'make beds'* for new admissions, creates *'pressure to rush the summary'* (consultant 2) for the authoring junior doctors, who will have *'a whole lot of other things to do'* (consultant 3). All secondary care clinicians acknowledged this reduced the quality of communication and ongoing care for the patient, and both GPs and GP registrars commented that it was noticeable on the receiving end:

*'* *You're constantly getting lots of demands for your attention, nurses want your discharge summary to be written quickly, you've got a patient to review on the ward that’s clinically urgent* *.'* (FY1 doctor 1)

The generally high workload facing GPs was appreciated by all focus group participants, and it was agreed that *interpretation* of any discharge summary should ideally take as little time as possible. GP 1 admitted that the time pressures also impact their performance in interpretation, and that key information could be missed if it was unclear, or required *'unpicking'*. If information is deemed to be missing, *resolution* via a telephone call or letter further adds to the GPs workload, and delays the decision that the response would support. While letters can be written to a consultant, response timeframes are uncertain. If the matter is urgent enough to require a telephone call, GPs described great difficulty in contacting a clinician who could answer their questions. Secondary care participants corroborated this and freely acknowledged that they can be a *'hassle to get hold of'* (consultant 3):

*'* *I don't even try. No. I just haven't got the time to sit on the phone for hours, going through "oh he’s not working today, he’s not here, no one remembers him, call back later".* *'* (GP 2)

The difficulty and time cost of pursuing *resolution* could become prohibitive in the context of GPs’ wider workload, particularly if needed for several patients. Giving feedback to discharge summary authors in the hope of future improvement was also described to be excessively time consuming and therefore often avoided:

*'* *You can't write back from every letter to the consultant to say "are you doing this?" or "has this patient definitely got an appointment?”* *.'* (GP 1)

#### Patient complexity

Although often without defining the term, the complexity of the patient’s case was described by all participants as a key determinant of the size and complexity of the communication system. There was consensus that as comorbidity, case complexity, or the need for multidisciplinary care increases, the difficulty of both *creation* and *interpretation* increases:

*'...* *the more complex someone is, the harder it is to write a decent discharge summary* *.'* (Consultant 1)

Complex patients may have larger quantities of more specialist information to curate, and can be less suitable to *'fit'* into standardised templates, even those that had been tailored to the particular specialties (FY1 doctor 2). Primary care participants indicated that complex cases increased the importance of adequacy and detail of the content, as they are more likely to need greater input, incur complications, and fall outside the area of the GP’s experience.

#### Attitudes and stakeholder relationships

Secondary care participants volunteered that discharge communication was not always as highly valued as it should be, and admitted that it can be seen as a *'boring, administrative task'* (GP registrar 2). This was seen as symptomatic of the *'disrespect shown to the profession of GPs within the NHS'* (FY1 doctor 2), and a lack of a ‘single team’ mentality emerged as a political undercurrent to the system. While moments of *'singing from the same hymn sheet'* (GP registrar 1) are cherished, the *'age-old divide between hospital and general practice'* (consultant 2) and the *'bigotry against primary care'* (GP 2) is ingrained. This was indicated to devalue discharge communication and to reduce efforts to maximise the quality of *creation*.

### What could go wrong? (Risk perspective)

The potential consequences of suboptimal system performance described by primary and secondary care stakeholders fell broadly into two categories.

'Hard events’ were those that could be identified as specific failures, including: the discharge summary never being sent; missing or unclear information; reader *interpretation* error; duplication of an investigation; avoidance or unsuccessful attempts to give feedback; use of *resolution* pathways by the GP; patient complaints; 'near misses'; direct patient harm; and unnecessary readmissions.

‘Soft effects’ of suboptimal communication were more subjective consequences that were harder to quantify, including: inefficient use of GP time and consequent detraction from care of other patients; reduced quality and continuity of care or poorer care coordination; increased uncertainty and risk for future decisions made by the GP; and a negative effect on GP–patient relationships.

### What does good look like? How can the needs be met? What could go wrong with the proposed solutions? (Design and risk perspectives)

Clinician stakeholder participants were asked what qualities they would envisage in a ‘good’ system, as well as possible improvement measures. Five main proposed ideas were discussed within the focus group and clinician interviews.

#### Formal teaching

The need for more formal teaching of junior doctors was described, and it was felt this would be most beneficial if delivered by a GP during junior doctors’ weekly teaching programmes. The possibility of discharge communication being incorporated into formative assessments was felt to be promising, especially if the assessment was done by a GP.

#### Formal feedback loop regarding quality of communication

Focus group discussion acknowledged the potential benefits of a facility to offer direct feedback to discharge summary authors and their teams, as *'the only way that you can fix things is if you know that they are good or bad*' (consultant 4). However, significant concerns were raised with respect to how the feedback would be received, particularly if it *'undermined'* the junior doctor authors, or if the teams disagreed over the nature of the feedback.

Given the volume of patients being discharged on a daily basis, the *'time factor'* (GP 2) involved with giving and handling feedback was felt be concerning, but avoiding feedback was not deemed to be acceptable when the patient perspective was considered.

#### Senior oversight for complex patients

While the addition to their workload was recognised, it was felt that the oversight of discharge communication by senior clinicians is essential as the patient becomes more complex, and to potentially be time-saving in the long run.

#### Direct email access between clinicians

Focus group participants acknowledged that the *creation* process would never meet its needs every time, and that therefore easy cross-interface communication was regarded as essential. This could be in the form of direct email contact, or through a messaging service within an IT system:

*'* *I mean* *,* *from my point of view, if I had their email address, I would be much more likely to ask questions and get responses* *.'* (GP 2)

The current lack of direct communication was felt to be detrimental to the atmosphere surrounding the primary–secondary care interface, and participants expressed that more direct communication would improve relationships and reduce *'animosity'* (GP registrar 3) across the interface:

*'* *I think they're much less likely to* *"* *bash* *"* *each other, if they're actually directly communicating* *.'* (GP registrar 2)

While the focus group participants unanimously anticipated that opening more direct lines of communication would ultimately be time-saving, it was acknowledged to have potential to increase workload and to be used excessively.

#### Shared records between secondary and primary care

The facility for *'everyone to be able to access each other’s stuff … just to know what’s going on'* (geriatric registrar), was unanimously considered to be positive during focus group discussion, and was also felt likely to improve the ‘team’ mentality of primary and secondary care. However, barriers to implementing this exist in the form of information governance and financial costs.

### Who should be involved?

The focus group participants were asked who would need to be involved to take any of the ideas any further, and five groups were identified: *'Clinicians, and from both sides'* (GP 2), with *'someone from at least every broad specialty'* (consultant 4) to maximise system coverage; undergraduate and postgraduate clinical education staff; GP liaison office; hospital and GP IT staff; and Patient Advisory Liaison Service to incorporate *'what patients are experiencing'* (GP registrar 2).

## Discussion

### Summary

A systems approach has provided a clear holistic understanding of the complex problem of discharge communication that has previously lacked definition.^6^ The system of communication studied was found to vary significantly between patients in terms of the number of interdependent elements, stakeholder needs, and the purposes of the communication. The more complex a patient’s care, the more complex the system, the higher the risks incurred, and the greater the difficulty of effective communication. Despite significant system variability between patients, the system’s structure as a largely one-way ‘open loop’^43^ presented a consistent hindrance to performance. The rarity of feedback and sharing of insights between stakeholders compounds a situation where primary and secondary care staff already lack an appreciation of each other’s perspectives and needs. A strong indication that this may lead to suboptimal patient care and increased risk was given. ‘Closing the open loop’ of the system therefore emerged as a key theme of how communication to GPs at hospital discharge might be improved. The measures proposed would facilitate broader system awareness and active sharing of performance insights between stakeholders, particularly for high-risk complex patients and scenarios, much as has been seen in other safety-critical industries.^44^

### Strengths and limitations

This research was designed as a scoping study of a wide and complex issue. A balance of depth and breadth of data was essential to build further ‘problem understanding’. The systems approach employed enabled this understanding to be developed, maintaining a necessary system-wide focus. Responders were drawn from a single hospital trust and surrounding geographical area in order to obtain rich data concerning one local information transfer system, seeking conceptual rather than statistical generalisation. Given that the PRSB template is a national standard,^28^ that junior doctors commonly move between hospitals, and that many of the findings are consistent with the existing literature, it appears that many of the findings are common across other settings.

The contextual data arising from the administrative and infrastructural staff interviews were not fully transcribed or formally thematically analysed. However, the detailed notes taken from repeated listening to the audio-recordings provided valuable context for emerging narratives and the development of a systems diagram (Figure 2), strengthening the value of the themes from the more formal thematic analysis of the clinician datasets and broadening the scope of the ‘problem understanding’ generated. Future work could usefully investigate these participants’ perspectives, alongside those of patients themselves.

Data collection was conducted largely by a clinician with experience of both primary and secondary care. Participants were aware of this, which may have limited their responses, although contrastingly they may have felt more comfortable discussing matters with an ‘insider’.^11^ The use of convenience sampling through professional contacts also gives potential for bias and participants may have had greater motivation to improve the system.

### Comparison with existing literature

The findings are consistent with existing literature, particularly in terms of the key role of discharge communication in patient safety^4,8–10^ and coordination of healthcare services,^45,46^ underlining the need for improvement in terms of quality and consistency.^6,13,29,47^ Further to this, a more holistic understanding of a typical NHS discharge communication system has been uncovered and drawn together, contributing clarity to the challenge of how discharge communication might be improved. This is the first study to apply a systems approach to discharge communication, and the findings regarding the relationship between complexity and the standardisation of communication are novel in this context. Standardisation has been described in the design literature as unsuitable for subjective and complex problems,^48^ with a risk of being 'misapplied' by users lacking expertise.^49,50^ Given this, it is unsurprising that encouraging discharge summaries to adhere to a standard^13,14,29^ has struggled to improve discharge communication to GPs, particularly for more complex patients. While the standardised template may not be ‘wrong’, for more complex cases it may misguide the author’s curation of information and impair the GP’s *interpretation*. All the potential discharge summary informational elements that participants outlined are reflected in the national PRSB standards,^28^ but given that recipient satisfaction depended more on the clarity and detail of each element, not simply on inclusion or exclusion, a key contribution from this work is that, instead of ‘answering’ a standard template, the *creation* process should be driven by the purposes of the communication foreseen for that patient. Information elements should then be selected and curated with a more bespoke ethos to serve these purposes, as illustrated in Figure 3.

### Implications for practice

Communication between primary and secondary care is likely to become increasingly strained as care becomes both more sub-specialised^51^ and yet increasingly shifts towards the community, as laid out in the NHS *L* *ong* *T* *erm* *P* *lan*.^52^ Given pre-existing safety concerns, inaction may have significant costs. Improvement could stem from GPs ‘closing the open loop’ of the system by taking a more active role in discharge communication, through formal teaching sessions for junior doctors, ideally held outside of the ‘overload’ of the induction period,^13^ and GP-led workplace-based assessments. The recently published discharge summary toolkit by the Royal College of Physicians^53^ has resources that could support this, particularly if informed by the purpose-driven approach that is proposed in this article. Awareness of the wider system structure and potential barriers to optimal communication, as outlined in Figure 4, should also be promoted.

The creation phase should be further supported by supervision of, and feedback on, discharge communication by senior hospital clinicians, particularly for complex patients. However, this should be corroborated by feedback from GPs to maintain insight into recipient perspectives. This process may be well supported by the future development of direct electronic messaging between primary and secondary care clinicians, a function that would also support *resolution*. Concerns understandably exist regarding time-costs and possible overuse of easier access communication, although a study of email communication in Scotland^54^ indicated that *'* *nipping things in the bud early* *'* was more time efficient in the long run, a sentiment shared by all participants of this study’s focus group. Shared electronic medical records, available in some areas of the UK, are a potential further support to resolution, although conversely they may result in information overload and failure to highlight key information and actions needed, and reduce the quality of creation.^16^ The NHS has a long history of failure to address the information technology, information governance, and financial issues involved with shared records, hindering widespread national rollout.

Future measurement of communication quality should relate to the meeting of articulated user needs rather than adherence to generic standards, and assess the message’s ability to serve purposes that are objectively foreseeable at the point of discharge, without incurring the need for *‘* *compensatory labour’* ^55^ via secondary care *resolution* pathways or unnecessary investigative work in primary care. Quality should be gauged from the recipient GP's perspective, and the authors recommend that the purpose-driven framework (Figure 3) forms the foundation of a more appropriate assessment, which could then be used to verify and validate any subsequent improvement measures. High-level organisations, such as the PRSB, should stress the pitfalls of standardisation and the importance of nuance in the context of high-risk complex patients, in future publications.
